# P04.60. The dutch complementary and alternative medicine (CAM) protocol: to ensure the safe and effective use of CAM within Dutch mental health care

**DOI:** 10.1186/1472-6882-12-S1-P330

**Published:** 2012-06-12

**Authors:** R Hoenders, M Appelo, E van den Brink, B Hartogs, J de Jong

**Affiliations:** 1 Center for Integrative Psychiatry, Lentis, Groningen, Netherlands; 2 Het Behouden Huys, Groningen, Netherlands; 3 University of Amsterdam, Amsterdam, Netherlands

## Purpose

Complementary and alternative medicine (CAM) is subject to heated debates and prejudices. Studies show that CAM is widely used by psychiatric patients, usually without the guidance of a therapist and without the use of a solid working method, leading to potential health risks. How can we use CAM alongside conventional psychiatry in an outpatient psychiatric clinic in a judicious and professional way?

## Methods

By searching through scientific and legal articles and discussion in focus groups a scientific model was formulated based on (1) patients’ needs and wishes; (2) respect for their freedom of choice; (3) a mix of western medicine and CAM that are safe and effective; (4) protection against quackery and abuse; (5) Dutch law, the jurisprudence of the Medical Disciplinary Tribunal and the rules of the Dutch Association of Medical Practitioners (KNMG) and (6) scientific evidence.

## Results

In the Centre for Integrative Psychiatry (CIP) of Lentis in the Netherlands some carefully selected CAM are offered under strict conditions, alongside conventional treatments. Because of the controversy and the potential health risks, Lentis designed a protocol which is presented. A clinical vignette is used to illustrate how this applies to daily practise.

## Conclusion

The Dutch CAM protocol provides a working method for the judicious use of CAM alongside conventional psychiatry in an outpatient psychiatric clinic.

